# Turkey’s Epidemiological and Demographic Transitions: 1931-2013

**DOI:** 10.4274/balkanmedj.2016.0960

**Published:** 2017-08-04

**Authors:** Coşkun Bakar, Sibel Oymak, Işıl Maral

**Affiliations:** 1 Department of Public Health, Çanakkale Onsekiz Mart University School of Medicine, Çanakkale, Turkey; 2 Department of Public Health, İstanbul Medeniyet University School of Medicine, İstanbul, Turkey

**Keywords:** Epidemiological transition, demographic transition, modernization, mortality data, Turkey

## Abstract

**Background:**

The causes of death have changed with regard to the epidemiological and demographic events in society. There is no evidence of prior research into the epidemiological transition in Turkey. This transition in Turkey should be observed starting with the Ottoman Empire period (19th to early 20th century). However, information about the Ottoman Empire is quite limited.

**Aims:**

To discuss the epidemiological and demographic transitions in Turkey, using demographic, educational and urbanization data in our present study.

**Study Design:**

A descriptive archive study.

**Methods:**

Mortality statistics dating from 1931 and published by the Turkish Statistical Institute were analysed, and the causes of death were coded and classified according to ICD-10. Other data were obtained from the published reports and studies regarding the issue.

**Results:**

In the 1930s, Turkey’s life expectancy was low (aged 40 years), fertility and mortality rates were high (respectively 45% and 31%), and the main causes of death were infectious diseases. Nowadays, life expectancy is close to 80 years, the total fertility rate has dropped to 2.1 per woman, and the main causes of death are chronic diseases and cancer. The population rate in the urban areas has increased steadily from 24.2% in 1927 to 77.3% in 2012. level of education has also increased during this period. In 1935, less than 10% of women were literate, and in 2013 90% were literate. Qualitative and quantitative increase have been observed in the presentation and access of healthcare services compared to the early years of the Republic.

**Conclusion:**

Turkey has been undergoing a modernization period in the last 200 years, and it is believed that the epidemiological and demographic transitions result from this period. This process has led to urbanization and an increase in the level of education, as well as a decrease in premature deaths, lower fertility rates, and an increase in the elderly population and chronic diseases. It is therefore our conclusion that Turkey needs policies regarding the elderly population and the management of chronic diseases.

“Epidemiological transition” is defined as the changes in the physical well-being of people that have been noticed, along with the demographic transition, since the Industrial Revolution (18^th^ century). Omran has defined three different models for epidemiological transitions: “*The Classical (Western) Model of Epidemiologic Transition”, “The Accelerated Epidemiologic Transition Model” and “The Contemporary (or Delayed) Epidemiologic Transition Model*”. The transition is characterized by the fact that degenerative and human-made diseases have replaced epidemics as the primary cause of morbidity and mortality. The theory starts with the affirmation that mortality is the principal factor in the dynamics of populations. The causes of death have changed with regard to the epidemiological and demographic events in a society. This informs how the demographic, economic and social dynamics factors that shape a society over time affect the understanding of health and illness (1,2,3).

There is no evidence of prior research into the epidemiological transition in Turkey. This transition in Turkey should be observed starting with the Ottoman Empire period (19^th^ to early 20^th^ century). However, information about the Ottoman Empire is quite limited. Nonetheless, the information we gathered through secondary sources shows that the Imperial Period corresponds to a pre-epidemiological period (4,5,6).

The aim of this study is to describe the epidemiological transition in Turkey between the years 1931 and 2013. The link between the epidemiological transition and the social dynamics is discussed in our study with the use of demographic, educational and urban transition data for the country for the same period.

## MATERIALS AND METHODS

### Study design

This research study is a descriptive archive study. In the methods section, first a short summary of the social history of the Republic of Turkey is presented to facilitate the understanding of the epidemiological transition. The results of the present study are evaluated and discussed through this process as well (This research was approved by the Çanakkale Onsekiz Mart University Clinical Research Ethics Committee on December 25, 2014, and the reference number is EK-2014-162).

### Study area: Republic of Turkey

The population of the Republic of Turkey was 76.667.864 in 2013, and 91.3% of the population were residing in urban areas (city or county centres) (7). The Turkish Statistical Institute (TSI) stated that 7.7% of the population was aged 65 or over, the annual population growth rate (PGR) was 13.7%, the crude birth rate (CBR) was 16.9%, the total fertility rate (TFR) was 2.1 per woman and the crude death rate (CDR) was 4.9%. In 2013, 357.581 deaths were reported by the TSI; 39.8% of these deaths were related to circulatory system diseases, 21.3% to cancers and 9.8% to respiratory system diseases (7,8,9,10,11).

### The pre-epidemiological period of transition (Pre-republic period: before 1923)

The Republic of Turkey (October 29, 1923) follows the Ottoman Empire, and has been going through a modernization period since the beginning of the 19th century. This period has accelerated with the transition to the structuring of a republic parallel to the reforms in the fields of education, health and industry. At the beginning of the 20^th^ century, when the Ottoman monarchic form of government was transformed into the Republic of Turkey, the majority of the population was living in the rural regions, the economy was based on agriculture and the level of education was low (6,12,13). In that previous Ottoman period, the high population in the rural regions, high death rates, infectious diseases and epidemics were important health concerns. Plagues were seen until the middle of the 19^th^ century, later followed by cholera epidemics, and the latter caused morbidity and mortality in the Republican period. In the early 20^th^ century, the main causes of death were infectious diseases, such as tuberculosis, and diseases presenting diarrhoea, typhus, cholera, smallpox, measles, diphtheria epidemics, rabies and trachoma (13,14,15,16,17). At the beginning of the 20^th^ century, the male population was dying at a young age because of wars. The fertility rate was on a downward trend in major cities. According to the results of the 1907 census, the TFR in İstanbul was nearly 3.5 and 7.0 throughout the Empire in relation to the pre-transition period (18,19). Since the second half of the 19^th^ century, preventive health services have been provided, and quarantine centres and local medical offices were founded as the first step of a health organization (15,20,21).

### The founding of the republic and the beginning of the epidemiological transition (1923-1960)

In 1923, the dynasty-based political organizational structure of the country was changed into one based on a republican structure and a parliamentary system. The new modality has set as its targets a nation state structure and modernization. The parliamentary republic is an important step for the democratization process. The Republic caused important developments in the economy, education and social life, and in the status and education of women. This period, when health data began to be monitored, is viewed as the beginning of the demographic and epidemiological transition. Especially after World War II, Turkey’s demographic and epidemiological transition profile began to evolve into a new era. Some important milestones of it include the following: after the Ministry of Health was established in 1930, a General Hygiene law was passed that can be considered revolutionary regarding preventive medicine and fighting against malaria, trachoma, tuberculosis and other infectious diseases (15,18,19,20,21,22). During this period, studies of the high infant and child mortality were carried out, and physicians, nurses, midwives and health-care officers were trained. Pro-natal policies were applied to deal with the population problem. This was an early transition period, when the TFR was between 6 and 7. The economy of the period was based on agriculture, with industrialization promulgated as an import substitution, and the creation of job opportunities was attempted. However, a significant proportion of the population was living in the rural areas. Urbanization is a concept that started to appear in Turkey only after the 1950s. In the studies that were carried out around 1945 and in the 50s, Ernst Reuter, Ömer Celal Saraç and Sadun Aren reached a common consensus that a powerful urbanization trend could not be observed in Turkey during that period. At that time, life expectancy was low and infectious diseases were still among the most important health issues (15,18,19,20,21,22).

### Welfare state policies, and an increase in chronic diseases (1960-1980)

The year 1960 was one of the most important turning points for the Republic of Turkey. A democratic experience that had just started to evolve was interrupted by the military coup of that year. After ratifying the new constitution in 1961, welfare state policies were preferred until the constitution of 1982, and as a result integrated health policies were applied to health services in which the rural area issues were prioritized. During this period, planned economic models were tried, urbanization and the level of education increased, the private sector grew, democracy continued to evolve and the European Union membership process began. Pro-natal policies were made more flexible in 1965. After this, family planning methods were allowed to enter the country. As a result of this, the TFR started to decrease.

Infectious diseases and infant mortality began to decrease, albeit slowly. Family planning services, mother and child health services monitoring children and pregnant women, and tuberculosis dispensaries spread across the country. In 1952, maternal and child health organizational studies started, in accordance with agreements made with the WHO and UNICEF. New programmes were adopted to fight against malaria, and health stations and primary health-care centres were opened to extend basic health-care services across the country (20,21,22,23). Vaccinations against smallpox, tuberculosis, diphtheria and polio were being used across the country. The TFR, which had been in decline since the 1950s, would not reach that same level thanks to the interventions during this period (4,5,19,24,25). In this same period, chronic diseases, mainly cardiovascular ones, began to take centre stage.

### Neoliberal policies period: chronic diseases and cancers, increase in life expectancy and in the elderly population (1980-2015)

In 1980, the Republic of Turkey entered a new era. With the impact of the crisis brought about by the economic and political instability of the 1970s and the international financial institutions, an export-oriented, liberal economic model was introduced to replace the intrusive and inward economic model that had been ongoing since the 1930s. The new model - also adopted by the military coup of 1980 - it forced a government restructuring. The liberal economy made downsizing of the government and the growth of the private sector necessary, and this has affected all segments of society, and especially so regarding the field of health. During this period, on the one hand, urbanization and the level of education increased in Turkey; on the other hand, there was unemployment, high inflation and problems owing to the imbalance in income distribution (19). An extended immunization programme and vaccination campaigns were introduced starting respectively in 1981 and 1985, and campaigns were held until the end of the 1990s. Smallpox was eradicated in the 1970s and polio cases have not been seen since 1998 in Turkey. Polio has not yet been eradicated in the world (15,20). Induced abortion until the 10^th^ week of pregnancy has been allowed since 1983 (26). There have been campaigns to increase women’s level of education. As a result of the latter, the literate population started to increase, and women were subsequently given a place in the workforce. The policies that were applied began to yield results in terms of demographic transition, early-mid transition (TFR: 5-5.9) in the 1960s, mid transition (TFR: 4-4.9) in the 1970s and late transition (TFR: below 3) in the 1990s (18,23).

The age of death has shifted from a young age to the age of 65. Fertility and mortality rates continued to drop, and since 2010 the TFR has dropped to the level of the renewal rate (TFR: 2.1). The late transition period has been reached in the fertility rate (TFR: 2.1-2.9) (18).

Structural changes in health care were made after the beginning of the year 2000. The basic health services organization that started as a pilot model throughout the country has been changed completely since 2011. According to this, at primary health-care services instead of a three-tiered health system (health station, primary health-care Centre and in district hospital), family medicine (individual health services) and community health centres (community health services) have been established, and the integrated health model that recommends both individual and community health services at the same unit has been discarded (23,24). The share of the private sector in the provision of health services has increased (7,27,28). To finance the service, a premium-based social insurance model was adopted. During this period, the Ministry of Health changed the definition of its services to the supervision and regulation of the service rather than service provision (23).

### Regulation of the data

### Mortality data

**Figure 1:**
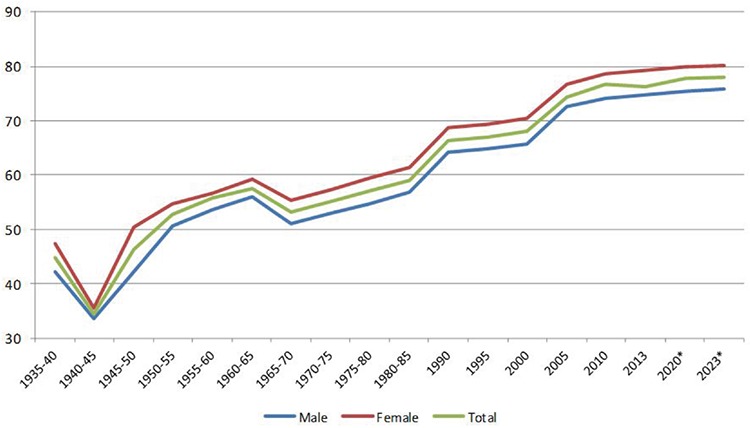
Life expectancies at birth according to years and gender (year), Turkey, 1935-2023. *Projection

**Figure 2:**
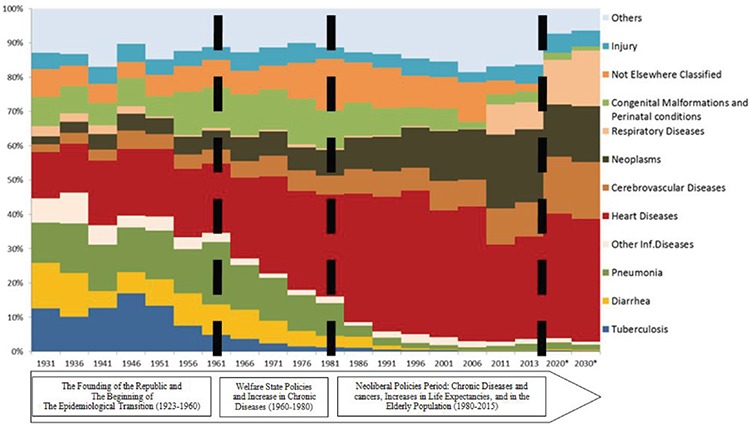
Distribution of causes of death according to disease groups and years, Turkey, 1931-2030. *Projection

**Figure 3:**
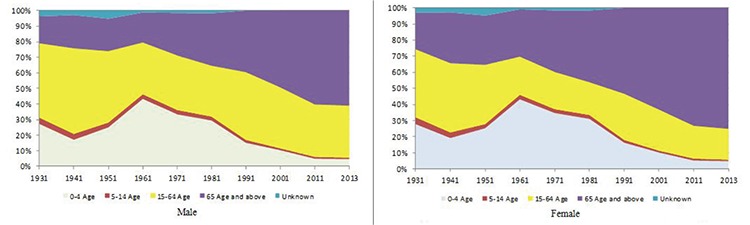
The distribution of ages of death according to gender, 1931-2013.

**Figure 4:**
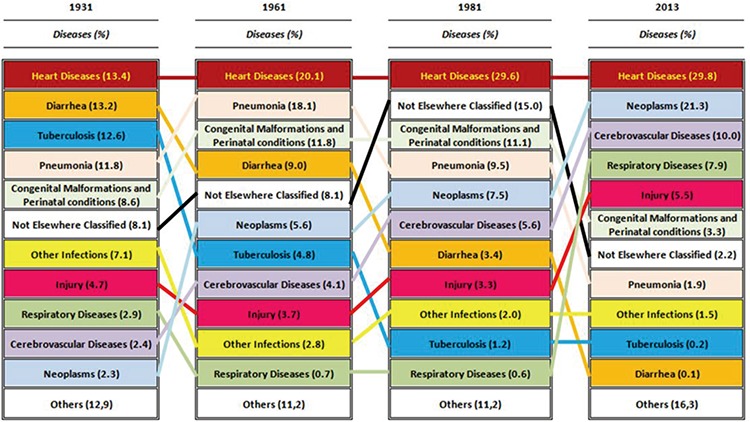
Transition of causes of death according to diseases, Turkey, 1931-2013.

**Figure 5:**
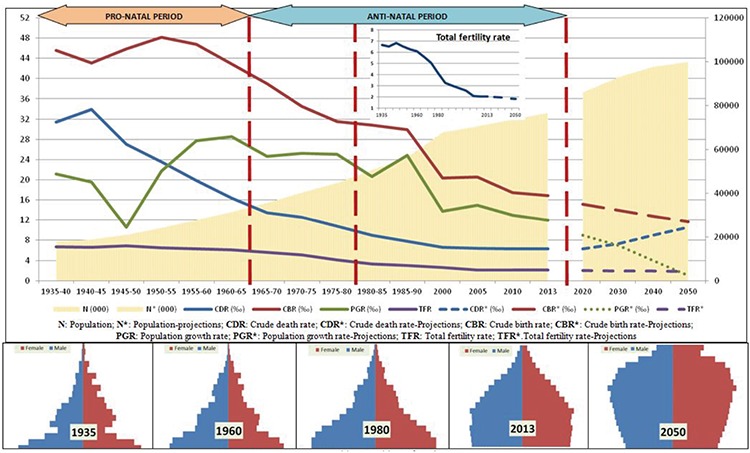
Demographic transition of Turkey 1935-2050.

Many sources have been used to obtain the research data, principally the TSI’s statistical yearbook. Mortality data were obtained from the statistical yearbooks that the TSI has been publishing since 1931 (Table 1). Statistics on causes of death began to be organized in 1931. For the statistics on the first 43 causes of death by diseases, List B - Abbreviated List of 50 Causes for the Tabulation of Mortality Converted from ICD-6 and ICD-8 was used (Table 1). Since 2009, the causes of death have been coded according to ICD-10. Statistics on death were collected from 25 provinces until 1949, and from 1957 mortality statistics began to be collected from all the provincial centres. Since 1957, death statistics have been collected from all provinces and districts (Table 1, Figure 1,2,3,4 and 5) (26,29,30,31,32).

Since 2001, a death notification system has been started electronically. Population records in Turkey have been kept electronically with a system called MERNIS since 2002. Thanks to this system, the TSI has been able to keep a record of population statistics since 2007. The TSI and the Ministry of Health renewed the death registration system to ensure compliance with the European Union in 2005. This new system has been used since 2009 (26,32,33).

The most important problem with the death data in Turkey is inclusiveness. According to the results of the National Burden of Disease and Cost-Effectiveness Project, Households Study (BoD-CE Project), published in 2004, 430.459 deaths in rural and urban areas were calculated. According to the projections made in this study, 500.307 deaths were projected for 2010. According to the TSI data, 185.141 deaths in 1999 and 174.315 in 2000 were reported. According to the WHO’s data, 426.100 deaths were reported in Turkey in 2013. The number of deaths reported by the TSI in 2013 was 357.581 (8,34,35,36). This leads us to infer that not all deaths are being registered, and the most important reason for this is thought to be the deaths in rural areas that can still not be accurately recorded.

### Life expectancy data

The data regarding life expectancy at birth in this study were taken from different sources: The “Second Five Year Progress Plan (1968-1972)” for the period until 1960-1965, the report “Turkey Health Statistics, of 2006”, prepared by the Turkish Medical Association for the period until 2000, the “2013 Health Statistics Yearbook” of the Ministry of Health for the period until 2013, and the report “The National Burden of Disease and Cost-Effectiveness Project, Households for the 2020 and 2030 Projections” (Figure 1) (7,28,34,37,38).

### Demographic, education and urbanization data

The following were used for the demographic data: the book *Population Growth in Turkey (1935-1975), Trends in Fertility and Mortality in Turkey;* the doctorate thesis by Türkay (39), entitled *Population Growth* and *Economic Development in Turkey;* a report on statistical indicators and population projections published by the TSI; Turkey’s “Demographic Transition” report published by Koç et al. (6); the publication “An Integrated Health/Family Planning Program in Etimesgut District, Turkey”, by Fişek and Shorter (4); “Turkey Health Reports” in 2000 and 2006 published by the Turkish Medical Association; the “Health Statistics Report 2013” published by the Ministry of Health; and the “Second Five Year Progress Plan (1968-1972)”. For the projections, the report “Correct Demography and Management towards 2050: Reflections on Education, Labor, Health and Social Security Systems”, published by the Turkish Industry & Business Association (TUSIAD), was used (Figure 5). Education and urbanization data were taken from the report “Statistical Indicators 1923-2012”, prepared by the TSI (4,5,6,7,8,25,28,39,40,41,42,43).

### Statistical analysis

The analyses and graphics research data were first transferred to excel electronically. The death statistics since 1931 from the TSI were published in the statistics yearbook presented in Table 1. Causes of death were published as a list of diseases with 43 items from 1931 to 1946, and a 50-item list from 1951 to 2006. The reports, except for the years 2001 and 2006, were published only as printed copies. The aforementioned reports are to be found in the TSI’s electronic library as PDF documents. Since the causes of death statistics since 2001 are to be found in the database of the TSI electronically, they were converted to Excel through this database. The causes of death since 1931 were first examined by the researchers on a yearly basis. To perform the analyses, they were transferred to Excel files in accordance with the tables in the reports, and manually by five-year intervals (1931, 1936, 1941,…2001, 2006, 2011, 2013). Following this, causes of death for each year were transferred and regrouped in accordance with the ICD-10 Main Disease Groups. For example, infectious diseases such as “typhoid and paratyphoid” and “measles”, which were among the causes of death in 1931, were coded in accordance with “Certain Infectious and Parasitic Diseases (A00-B99)”, the group that is in the ICD-10 Main Disease Groups. In this way, all the causes of death before 2011 were regrouped in accordance with the ICD-10 main disease group, with which it complies. The TSI began to publish the causes of death in accordance with ICD-10 after 2009; this is the main reason why the 2011 and 2013 data were taken directly from the TSI database in Excel format. The projections for the years 2020 and 2030 were taken from the National Burden of Disease and Cost-Effectiveness Project reports. After all the causes of death were classified according to the Main Disease Groups, the cause and age-specific death rates were calculated proportionally. Age-specific proportional death rates were also calculated separately for men and women (Table 1) (4,5,6,8,9,10,11,26,28,32,40,41,44,45). All data, except the mortality data, were directly transferred to excel documents manually, and the graphics were created in this program as well. This stage of the research was conducted in approximately four months between October 2014 and January 2015. Following this, the data obtained were interpreted and the study was written.

## RESULTS

This is the first study of the epidemiological transition of Turkey. Using death data, Turkey’s epidemiological transition was described, and demographic, educational, urbanization and social development factors of the country were discussed together. The change in life expectancy at birth can be seen in Figure 1. It tends to increase constantly, although it decreases between 1940 and 1945 (Figure 1).

In Figure 2, the transition of the causes of death in Turkey since 1931 can be seen. In 1931, 44% of the causes of death were infectious diseases, followed by circulatory system diseases at 16%, and congenital and perinatal causes at 8.6%. Cancers only had a 2.3% share in 1931. The most important causes of death were heart diseases, diarrhoea, tuberculosis and pneumonia (Figure 2, 4). One of the remarkable findings is that deaths owing to malaria were at 2.3%. A similar situation continued into 1936. While infectious diseases had a share of 46.4%, circulatory system diseases were at 17.5% (Figure 2). Death rates related to infectious diseases in this period, while the republic was newly founded, were at the forefront, and life expectancy was short and the fertility rates high.

The transition of the causes of death seems to be obvious, starting from 1951. The trend for circulatory system diseases, especially heart diseases, was to increase. The most important reasons for infectious diseases decreasing in number were diarrhoeal diseases and tuberculosis. Infectious diseases were never at the top of the list after the 1960s. Deaths by congenital malformations and perinatal causes entered a continuous downward trend after the 1980s, after remaining at around 10% in the 1960s. Cancer-related deaths initially had a share of 2%; however, later they started to increase. This increase, though, was not very apparent until the 1980s, and was, at most, 8%. After the 1990s, cancer-related deaths exceeded 10%. In the 2000s, cancer-related deaths became the second most important cause of death (Figure 2, 4).

In 1931, a significant proportion of deaths was seen in the 0-4 and 15-65 age groups. In 1961 in particular, 40% of the deaths were in the 0-4 age group. In 2013, 70% of the deaths of women and 60% of those of men were of people aged 65 or older (Figure 3).

Between 1940 and 1945, the CDR increased and the CBR decreased in the country. Later, until 1950-1960, the CBR and TFR continued to increase and began to continuously decrease after that. Since the 1940s, the CDR has continued to decrease until now. The PGR, although showing fluctuations, increased until the 1960s-1970s and decreased later on. The population of the country increased from 13 million to 76 million in this period (1923-2015). According to the projections by the TUSIAD, the population of the country will continue to increase to 95-100 million until 2050. According to the projections the TSI has made with different scenarios, the population is expected to exceed 100 million (9,40,43). The demographic transition trend of the Republic of Turkey started in the 1930s, and reached its final phase with low birth and death rates (Figure 5).

In 1935, less than 10% of women were literate, and in 2013 90% were literate. Also, the number of women and men with graduate degrees from universities increased during this period. In 1935, 167 women and 711 men graduated from a higher education institution. In 2011-2012, these figures reached 270.120 and 303.314, respectively. The population rate in the urban areas has increased steadily (urban areas, 1927, 24.2%). As of 2012, 77.3% of the population lived in urban areas (40).

## DISCUSSION

This first study to interpret the demographic and social transitions in Turkey together presents important information. Life expectancy, except for the years 1940-1945, has generally been on an increasing trend. The 1940-1945 period corresponds to World War II. The decrease in life expectancy shows that, despite not participating in the war, the country was affected by it negatively (Figure 1). Life expectancy in the world shows a continuously increasing trend, especially in the last 25-30 years (46,47,48). This increase has been similar in our country; however, this trend has also resulted in an increase of the elderly population. The elderly population rate (ages 65 and over) had a share of 7.7% in 2013, and is expected to be 20.8% in the year 2050, and 27.7% in 2075 (11).

The transition in Turkey fits “*The Contemporary (or Delayed) Epidemiological Transition Model*” that Omran (3) defined. In this model, the transition in developing countries such as Chile and Ceylon began slowly and hesitantly at the beginning of the last century and accelerated after World War II. The measures taken in public health and the programmes implemented under the sponsorship of international organizations affected the decrease in fertility (3). Turkey fits more in this latter group in both the demographic and epidemiological transitions. The main driving force for the transition in our country is the modernization process that started in the 19th century. The pro-natal policies applied in the early years of the republic were replaced by anti-natal policies after the 1960s. Between the years 1970 and 1990, institutions such as the WHO and UNICEF supported these policies. Policies aimed at preventing the growth of the population began to yield results after the 1970s, but the potential growth of the population has continued until today, and is expected to continue in the coming years (it is estimated that the population, currently around 76 million, will increase to the level of 90-100 million and then will become stable) (Figure 5) (6,15). From this perspective, it can be said that the demographic and epidemiological transitions began in our country early in the 20th century and accelerated after the 1960s. Life expectancy continues to increase and the elderly population is on an upward trend. Unless there is a pause or a regression in the demographic transition, fertility rates will continue to fall.

In the 1930s, tuberculosis, pneumonia and diarrhoeal diseases were among the leading causes of death. During the same period, malaria also stood out as a cause of death. Today, malaria is under control and the number of cases is very low (13,15,21). In the 1960s, deaths due to tuberculosis, pneumonia and diarrhoeal diseases began to decrease, and deaths owing to heart diseases started to increase. An increase appears in the statistics for cancers in the 1980s and for respiratory system diseases in the 2000s. This change in the respiratory system diseases is estimated to be related to the coding systems. Nevertheless, deaths due to respiratory system diseases have increased in our country in recent years (7,28,49). Cancer diseases as a cause of death reached 10% in the late 1980s, and today are around 20%. There are other studies that conclude that cancers take second or the third place among the causes of deaths. The factors contributing to cancer being among the important causes of death are smoking, drinking, obesity and eating habits, as well as pollution in industrial areas (50,51,52,53,54,55). Atun et al. (23), in their “Global Burden of Disease 2010 Analysis”, published in 2013, stated that the chronic disease burden increased by 50% in terms of disability-adjusted life years (DALYs) between the years 1990 and 2010. Ischaemic heart diseases, cerebral-vascular diseases, major depressive disorders and cancers will be among the important disease burdens. Public health programs need to be implemented to protect the public from these diseases. Some programmes are conducted by the Ministry of Health (23).

The first years of the republic, dominated by pro-natal policies, correspond to the periods we call “pre-transition” and “early transition” stages. The most important feature of this period is that the economy was based on agriculture. With the import substitution policies implemented in the 1930s, it was possible to create new job opportunities. More land was opened for agriculture, therefore migration to urban areas from rural areas was not an issue. Until the 1930s, the urban population remained below 30%, and, as previously stated, strong urbanization trends were not observed (18,22). In this period, death rates at young ages were high, and the literacy rate, especially in women, was low. Pro-natal policies implemented during this period yielded some results: the TFR increased up to 7 in the early 1930s and in the 1950s (Figure 5) (6,18). After World War II, thanks to the social and economic transition in the country, the extension of the health policies to include rural areas and the initiation of the family planning services, families began to have the number of children they planned, with fewer births, and the birth rate started to fall. According to the assessment carried out by Pamuk (19), the cause of this situation resulting in the decrease of the fertility rate was not the population planning programmes, as claimed by the politicians, but rather the conscious choice of married couples guided by economic, social and cultural developments.

Figure 3 shows that the mortality rate in the 0-4 age group started to increase between the 1950s and 1960s. There is no evidence to explain what caused this. Based on the data we have, it is believed that it may have been related to the decline in deaths in the 15-64 age group between the 1940s and 1960s. This could have been expected to have affected the 65 years and over age group. However, it is thought that the increase in deaths during young age until the 1970s could have limited this increase. Nevertheless, there was a slight increase in the number of deaths of people over the age of 65 between 1950 and 1960, especially in women (Figure 3). Figure 3 shows that between 1950 and 1960 the death rate in the 0-4 age group was beginning to increase. However, there is no evidence to explain what caused this, and it is thought that this increase may have been related to the decline in deaths in the 15-64 age group between 1940 and 1960. This could have been expected to have affected the 65 years and over age group. Nevertheless, it is thought that many deaths during young age by the 1970s could have limited this increase.

A downward trend in mortality began in the 1970s. However, this decrease, especially in the infant mortality rate, was not rapid. Until the late 1990s, infant mortality rates only fell to a mere 40 in a thousand. The low level of women’s education in the eastern part of the country and in the rural areas, the low social status of women and the inequalities owing to regional differences were among the reasons for this slow decrease (19,56). It has decreased to 13 in a thousand in the last 15 years, and infant deaths were reduced by two-thirds between 1990 and 2015, which was one of the Millennium Development Goals and was achieved in Turkey (44). The most important driving force behind this is the decrease in death rates caused by pneumonia, diarrhoeal diseases and tuberculosis (Figure 2, 4).

The estimates made so far show that the fertility rate is going to continue to decrease but the growth of the population will also continue owing to the potential caused by the fertile population (Figure 5) (6). If such trends continue, the post-transition stage will be reached in the next period (TFR: 0-2.0) (18). In it, the young population will show a decreasing trend, and in time it will be replaced by the elderly population. Nonetheless, as a result of the fertility rates of the past, the working age population will grow. This phase, which was called a “Window of Opportunity” by Barlow, is estimated to continue for approximately 25-30 years. At this point, population policies should be discussed not in terms of their quantitative but their qualitative aspects (6,18). On the other hand, according to the Turkish Population and Health Research results, the TFR is on the increase by 2.26 (44). It is still too early to tell whether the increase is a sign of a future increase in the fertility rate. For this, fertility features in different groups must be discussed. However, the attitudes and practices of the current political powers that encourage a population increase may mean that this is not a remote possibility.

Epidemiological and demographic transitions are historical facts that coexist under the influence of the economic, social and cultural transitions of a society. The epidemiological and demographic transitions in Turkey started in the early 20^th^ century with the modernization trend inherited from the Ottoman Empire, the poor economic conditions owing to long-running wars, the high population in the rural areas, the low level of education, young deaths, infectious diseases and the high fertility heritage. In the 1950s, the urbanization rate and the education level increased. With the social transition that took place in this period, first cardiovascular diseases and then cancers replaced infectious diseases statistically. Life expectancy at birth increased during this time. Also, pro-natal policies were abandoned and family planning services were launched. Health care started to spread across the country with the Basic Health Care approach 245/5000.

The socialization practice of health services implemented after 1960 in our country has made it possible to expand primary health-care services throughout the country, especially in rural areas through health centres/health stations. These institutions have been able to deliver basic health services, such as anti-natalist policies and immunization programmes, to the whole of Turkey. The health-care system has been through important changes over the past 15 years. There have been quantitative increases, but the integrated health services with the Basic Health Care approach were mainly divided into two, individual and society-oriented services. Political will has led to efforts to return to the pro-natal policies for economic reasons.

The elderly population in particular has increased significantly in the last 20 years, and its priority health issues have been circulatory system diseases, cancers and other chronic diseases. As a result, the most important problem in Turkey now and in the future is with the elderly population with chronic health problems. Until now, the society has tried to take care of its few elderly people with a traditional approach; however, today this approach cannot solve any problems. New approaches are needed regarding services for the elderly. The priority should be to keep the elderly more active in social life, since this is thought to reduce their dependence process. Health care should be planned according to the particular elderly people and to the priorities of the chronic diseases at all stages. The costs for these services cannot be afforded by everyone in the community. The government should allocate funds that are distributed in relation to the non-fatal disease burden. The allocated funding should be used for at-home or institutional health-care services that do not include medical treatments. In order to facilitate access to health services, primary health-care services should be strengthened and policies should be implemented that encourage all groups in the community to use these services first. Furthermore, all common living areas and houses should be designed so that the elderly and the handicapped do not become detached from social life.

## Figures and Tables

**Table 1:**
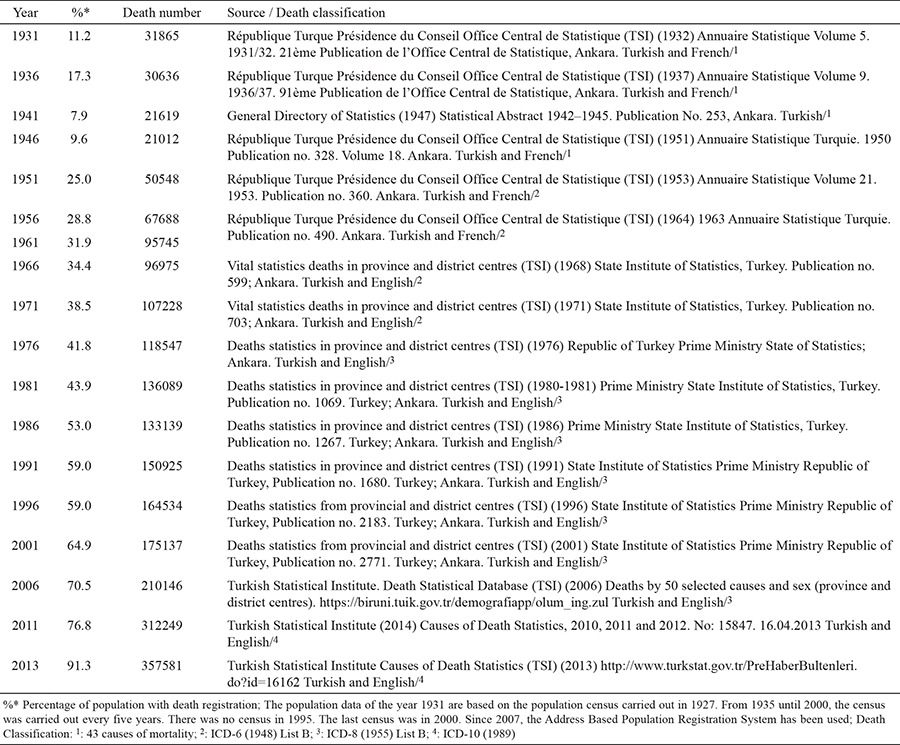
Sources of data used for evaluating death statistics, Turkey, 1931-2013
